# Alkamides from *Anacyclus pyrethrum* L. and Their in Vitro Antiprotozoal Activity

**DOI:** 10.3390/molecules22050796

**Published:** 2017-05-12

**Authors:** Julia B. Althaus, Claudine Malyszek, Marcel Kaiser, Reto Brun, Thomas J. Schmidt

**Affiliations:** 1 Institut für Pharmazeutische Biologie und Phytochemie (IPBP), University of Münster, PharmaCampus, Corrensstraße 48, Münster D-48149, Germany; julia.althaus@uni-muenster.de (J.B.A.); cmalyszek@brial.com (C.M.); 2 Swiss Tropical and Public Health Institute (Swiss TPH), Socinstraße 57, Basel CH-4002, Switzerland; marcel.kaiser@unibas.ch (M.K.); reto.brun@unibas.ch (R.B.); 3 University of Basel, Petersplatz 1, Basel CH-4003, Switzerland

**Keywords:** *Anacyclus pyrethrum* L., antiprotozoal activity, alkamide, *Plasmodium falciparum*, *Trypanosoma brucei rhodesiense*, *Trypanosoma cruzi*, *Leishmania donovani*

## Abstract

In our ongoing study to evaluate the antiprotozoal activity of alkamides from Asteraceae, a dichloromethane extract from the roots of *Anacyclus pyrethrum* L. showed a moderate in vitro activity against the NF54 strain of *Plasmodium falciparum* and against *Leishmania donovani* (amastigotes, MHOM/ET/67/L82 strain). Seven pure alkamides and a mixture of two further alkamides were isolated by column chromatography followed by preparative high performance liquid chromatography. The alkamides were identified by mass- and NMR-spectroscopic methods as tetradeca-2*E*,4*E*-dien-8,10-diynoic acid isobutylamide (anacycline, **1**), deca-2*E*,4*E*-dienoic acid isobutylamide (pellitorine, **2**), deca-2*E*,4*E*,9-trienoic acid isobutylamide (**3**), deca-2*E*,4*E*-dienoic acid 2-phenylethylamide (**4**), undeca-2*E*,4*E*-dien-8,10-diynoic acid isopentylamide (**5**), tetradeca-2*E*,4*E*,12*Z*-trien-8,10-diynoic acid isobutylamide (**6**), and dodeca-2*E*,4*E*-dien acid 4-hydroxy-2-phenylethylamide (**7**). Two compounds—undeca-2*E*,4*E*-dien-8,10-diynoic acid 2-phenylethylamide (**8**) and deca-2*E*,4*E*-dienoic acid 4-hydroxy-2-phenylethylamide (**9**)—were isolated as an inseparable mixture (1:4). Compounds **3**, **4**, and **5** were isolated from *Anacyclus pyrethrum* L. for the first time. While compounds **4** and **5** were previously known from the genus *Achillea*, compound **3** is a new natural product, to the best of our knowledge. All isolated alkamides were tested in vitro for antiprotozoal activity against *Plasmodium falciparum*, *Trypanosoma brucei rhodesiense*, *Trypanosoma cruzi*, and *Leishmania donovani* and for cytotoxicity against L6 rat skeletal myoblasts.

## 1. Introduction

Alkamides represent a class of natural products with considerable structural diversity and a broad spectrum of bioactivities [1]. In a previous study, alkamides from *Achillea ptarmica* L. (sneezewort yarrow, Asteraceae) showed activity against *Plasmodium falciparum* (*Pf*) and *Trypanosoma brucei rhodesiense* (*Tbr*), causative agents of tropical malaria (*Pf*) and human African trypanosomiasis (HAT, “Sleeping Sickness”; *Tbr*), respectively. However, the activity of the isolated alkamides could not conclusively explain the high activity of the tested dichloromethane extract against *T. b. rhodesiense* (IC_50_ 0.67 µg/mL) [2]. Recently, an alkamide structurally related to those found in *A. ptarmica* but with an endoperoxide structure was isolated from *Acmella ciliata*, (also Asteraceae) and showed considerable antiplasmodial activity against *Pf* [3].

These studies on alkamides from Asteraceae were extended to *Anacyclus pyrethrum* L. and its alkamide constituents. From a phylogenic point of view, *Anacyclus pyrethrum* is closely related to *Achillea ptarmica* L. within the tribe Anthemideae of Asteraceae [4,5,6], and both species are known to contain a structural pattern similar to alkamides [7]. *A. pyrethrum* has been known since ancient times for its use as medicinal plant used—among other ailments—against recurring fevers (*De Materica Medica* [8]), and it was mentioned in a review by Adams as one of the plants used against malaria during the Renaissance (16th and 17th century) [9]. In a bioactivity study associated with the mentioned review, antiplasmodial activity was reported for an ethyl acetate extract of *A. pyrethrum* roots, but without mentioning the level of activity in terms of an IC_50_ value and without specification of the active constituents [10]. We recently reported on the antiprotozoal IC_50_ values of a dichloromethane extract from this plant drug, including a value of 3 µg/mL for the activity against *Plasmodium falciparum* [2].

The aim of the present study was therefore to evaluate the antiprotozoal activity of the alkamides in the roots of *A. pyrethrum*.

## 2. Results and Discussion

### 2.1. Isolation and Structural Characterization of the Alkamides

**Figure 1:**
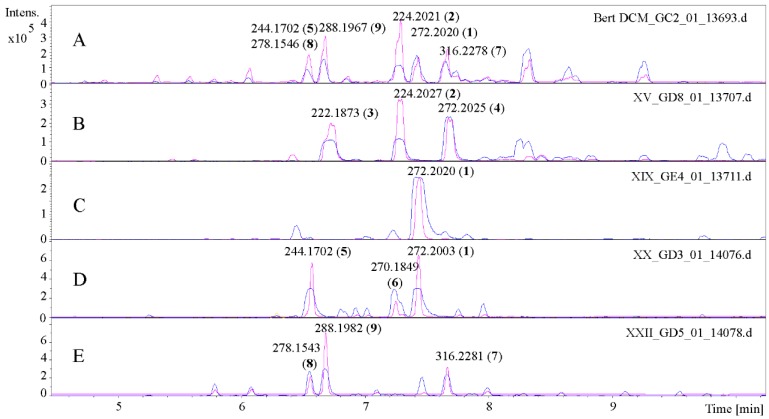
UHPLC/+ESI QqTOF MS-MS chromatograms of the CH_2_Cl_2_-extract of *Anacyclus pyrethrum* L. (**A**) c = 10 mg/mL and the selected fractions (**B**–**E**) c = 5 mg/mL. (**B**) fraction XV, (**C**) fraction XIX, (**D**) fraction XX, and (**E**) fraction XXII. All peaks are labelled with the measured *m*/*z* values and compound numbers. Blue represents base peak chromatogram *m*/*z* 100–500, and magenta represents base peak UV-chromatogram at 260 nm.

The major alkamides of A. pyrethrum roots were isolated from the dichloromethane extract by column chromatography (CC) on silica with an n-hexane-ethylacetate gradient followed by preparative high performance liquid chromatography (HPLC) of representative CC-fractions (XV, XIX, XX and XXII) selected on the basis of their UHPLC/+ESI QqTOF MS chromatograms (see Figure 1).

The alkamides were identified by their exact molecular masses determined by UHPLC/+ESI-QTOF-MS/MS and by NMR spectroscopy.

**Figure 2:**
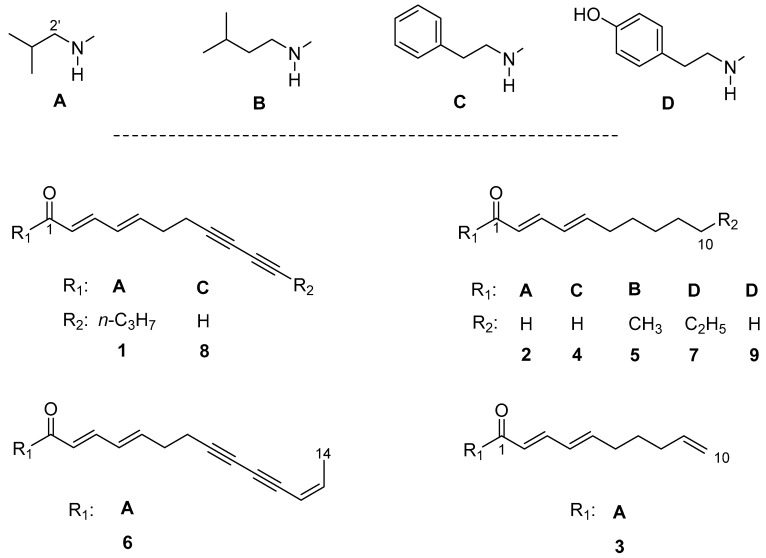
Structures of the isolated alkamides from *A. pyrethrum*. Deca-2*E*,4*E*,9-trienoic acid isobutylamide (9,10-dehydropellitorine; compound **3**) is a new natural product.

The main component of fraction XIX (Figure 1, chromatogram C) with *m*/*z* 272.2020 [M + H]^+^ was identified by its mass and NMR data as tetradeca-2*E*,4*E*-dien-8,10-diynoic acid isobutylamide (anacycline, **1**) in direct comparison with the compound previously isolated from *A. ptarmica* and literature data [2,11]. Three compounds were isolated from fraction XV (Figure 1, chromatogram B). Compound **2** (*m*/*z* 224.2027 [M + H]^+^) was identified (in the same manner as **1**) as deca-2*E*,4*E*-dienoic acid isobutylamide (pellitorine) [2,12]. Compound **3** (*m*/*z* 222.1873 [M + H]^+^) displayed a similar ^1^H-NMR spectrum as **2**, but with signals typical of a pair of germinal olefinic protons at position C-10 (Figure S1). All proton resonances were assigned to the corresponding ^13^C-NMR signals (Figure S2) by an HSQC experiment (Figures S3 and S4), and the ^1^H/^1^H COSY as well as ^1^H/^13^C HMBC spectra (Figures S5–S7) allowed unambiguous assignment of structure of **3** as deca-2*E*,4*E*,9-trienoic acid isobutylamide (9,10-dehydropellitorine; Figure 2)—a new natural product to the best of our knowledge.

A further compound with a molecular mass of 272 amu ([M + H]^+^ at *m*/*z* 272.2025) was identified as deca-2E,4E-dienoic acid 2-phenylethylamide (**4**) by its ^1^H-spectrum (Figure S8) in comparison with literature [13]. The compound was known from *Achillea wilhelmsii* but is now described as a constituent of *A. pyrethrum* for the first time.

Fraction XX (Figure 1, chromatogram D) contained—besides further anacycline (**1**, *m*/*z* 272.2003 [M + H]^+^)—two further compounds which could be identified as undeca-2*E*,4*E*-dien-8,10-diynoic acid isopentylamide (**5**, *m*/*z* 244.1702 [M + H]^+^) and tetradeca-2*E*,4*E*,12*Z*-trien-8,10-diynoic acid isobutylamide (**6**, *m*/*z* 270.1849 [M + H]^+^). Compound **5** was identified by comparison of its NMR spectra (Figure S9), which were in full agreement with literature data [11] reported after isolation from *Achillea ptarmica* L. This is the first report on the occurrence of **5** in *A. pyrethrum*. In the case of compound **6**, it was reported in the literature that the all-E-isomer (tetradeca-2*E*,4*E*,12*E*-trien-8,10-diynoic acid isobutylamide) is a constituent of *Anacyclus pyrethrum* L. [1,14]. In our investigation, the isolated compound was unambiguously found to be the Z-isomer with regard to the C-12-C-13 double bond, since the coupling constant of H-12 and H-13 was 11 Hz. Since no NMR data proving the double bond geometry were reported in the original literature [14], it cannot be excluded that the reported constituent of A. pyrethrum was identical with compound **6**. It could be possible, however, that both isomers occur in the plant.

In fraction XXII (Figure 1, chromatogram E), three compounds (**7**–**9**) with [M + H]^+^ adduct ions at *m*/*z* 316.2281, 278.1543 and 288.1982, respectively, were isolated, with the latter two in a mixture of approximately 1:4. Their NMR spectra in comparison with literature [15] allowed unambiguous identification as dodeca-2*E*,4*E*-dien acid 4-hydroxy-2-phenylethylamide (**7**), undeca-2*E*,4*E*-dien-8,10-diynoic acid 2-phenethylamide (**8**), and deca-2*E*,4*E*-dienoic acid 4-hydroxy-2-phenylethylamide (**9**), respectively, all known constituents of *A. pyrethrum* [15].

### 2.2. Antiprotozoal Activity of the Isolated Alkamides

The IC_50_ values for the in vitro antiprotozoal activity against *Trypanosoma brucei rhodesiense* (*Tbr*), *Trypanosoma cruzi* (*Tc*), *Leishmania donovani* (*Ld*) and *Plasmodium falciparum* (*Pf*), as well as the cytotoxicity against L6 rat skeletal myoblasts of the crude dichloromethane extract from *A. pyrethrum* roots and of anacycline and pellitorine (**1** and **2**) were tested earlier under identical conditions, and the results were reported in our earlier study [2]. The respective data of the isolated compounds **3**–**7** as well as the 1:4 mixture of **8** + **9** were determined in this study and are reported in Table 1.

On the background of the reported medicinal use as an antimalarial [8,9], it appears noteworthy that the crude extract displayed its highest activity against Pf, even though the IC_50_ value of 3 µg/mL is relatively moderate. The most active single constituent against this parasite is compound **7** with an IC_50_ in the same range (3.2 µg/mL), and about equal to that of the pellitorine (**2**, 3.3 µg/mL) [2]. Compound **7** was also found to be the most active compound against Tc. Unfortunately, however, the antiparasitic activity of ***7*** is not selective. The compound, as the only one in the series, displayed considerable cytotoxicity with an IC_50_ of only 0.2 µg/mL, so further studies on its antiprotozoal activity do not appear to be useful. The mixture of **8** and **9** displayed some activity against Tbr, but with little selectivity. The major component in this mixture (**9**) is structurally very similar to **7**, and hence is likely to dominate the observed antitrypanosomal as well as cytotoxic activity. The ratio of cytotoxic over antitrypanosomal activity being more favorable for the **8** + **9** mixture than for **7**, it could be expected that **9** with its C_10_ acyl moiety, as a pure compound, would be more active and selective against *Tbr* than its C_12_ homologue **7**. In terms of structure–activity relationships, the *p*-hydroxy-substituted phenethyl amide moiety of these two compounds appears to confer higher toxicity to trypanosomatid as well as mammalian cells than the unsubstituted phenethyl amide moiety or the aliphatic amide groups. The other compounds showed only moderate activity against all tested parasites, and also low cytotoxicity. No clear-cut structure–activity relationships became evident for these compounds.

Taken together, the two alkamides **2** and **7** are likely to be responsible for the major part of the antiplasmodial effect of the crude extract, and thus related to the ethnomedical use of *A. pyrethrum* as an antimalarial. It must remain an open question at present whether this use was justified and whether, possibly, other compounds from yet-untested fractions contribute to the overall effect.

## 3. Materials and Methods

### 3.1. Analytical Procedures and Instruments

#### 3.1.1. UHPLC/+ESI-QTOF-MS/MS

The analytical profiling of samples were performed on the already-described system with the identical method [2].

Sample concentration: concentration of pure compounds: 0.1 mg/mL (**1**–**7**) or 0.2 mg/mL (**8** + **9**) in methanol; concentration of fractions: 5 mg/mL; concentration of crude extract: 1 or 10 mg/mL.

#### 3.1.2. NMR Spectroscopy

Nuclear magnetic resonance spectra were recorded on an AS 400 Mercury plus spectrometer (Varian, Palo Alto, CA, USA) at 400 (^1^H, **5** and **6**), 600 (^1^H, **1**–**4** and **7**–**9**) and 100 MHz (^13^C) CDCl_3_ at room temperature. They were referenced to the solvent signals (δ_H_ 7.260 ppm, δ_C_ 77.000 ppm).

### 3.2. Isolation Process

#### 3.2.1. Plant Material

The plant material (roots of *Anacyclus pyrethrum* L.; latin description: Pyrethi radix; specification: “*scharf*” (= “spicy”), powdered) was purchased from Alfred Galke (Bad Grund, Germany). A voucher specimen (# TS_Apy_01) is deposited at the IPBP herbarium.

#### 3.2.2. Sohxlet Extraction

The powdered plant material (604 g) was exhaustively extracted in two equal portions with dichloromethane (1500 mL each) in a Soxhlet apparatus for 11 h. The extract was evaporated to dryness under reduced pressure, yielding 14 g of crude extract.

#### 3.2.3. Gravity Flow Column Chromatography (CC)

The extract (12.95 g) was separated by CC on 1.2 kg silica (particle size 0.063 to 0.2 mm; Merck, (Darmstadt, Germany), column dimensions 108 cm × 6 cm). The column was covered with aluminum foil to prevent the light-induced polymerization of alkamides with a polyinic structure moiety [16]. The silica was equilibrated at 90:10 *n*-hexane/EtOAc (2.4 L). The flow was adjusted to 1 mL/min, and 10 mL were collected per tube for the first 459 tubes. From tube 460 to tube 2023, 20 mL were collected per tube. A gradient with an increasing amount of EtOAc was used: *n*-hexane/EtOAc 90:10 (3 L); 80:20 (1.7 L); 70:30 (6.8 L); 60:40 (1.7 L); 50:50 (1 L); 40:60 (3.7 L); 30:70 (1 L); and 20:80 (3.7 L). Finally, 3 L of isopropanol was used to obtain the most polar fraction. Related eluates were combined after TLC control (silica gel 60 F_254_, Merck (10 cm × 20 cm); detection: anisaldehyde/sulfuric acid, UV 254 nm, 366 nm and daylight; elution: current solvent mixture of the CC column). The fractionation is summarized in Table 2.

#### 3.2.4. Purification by Preparative High Performance Liquid Chromatography (HPLC/UV-DAD)

The preparative HPLC isolation of all alkamides was performed on a Jasco (Groß-Umstadt, Germany) prep. HPLC system (pump: PU-2087 plus; diode array detector MD 2018 plus; column thermostat CO 2060 plus; autosampler AS 2055 plus; LC Net II ADC Chromatography Data Solutions; sample injection loop: 2000 μL) on a Reprosil 100 C-18 column (5 μm, 250 mm × 20 mm) column (precolumn: C-18, 5 μm, 30 mm × 20 mm) with a binary gradient (A: water (Millipore); B: acetonitrile (HPLC grade)) at 15 mL/min with: 0 to 5 min: linear from 40% B to 65% B; 5 to 30 min: isocratic 65% B; 30 min to 40 min: linear from 65% B to 100% B; 40 min to 50 min: isocratic 100% B. The subfractions XIX and XV were each dissolved in a concentration of 10 mg/mL acetonitrile and injected in portions of 400 μL. The subfractions XX and XXII were dissolved in a concentration of 10 mg/mL acetonitrile/water (70:30) each and injected in portions of 400 µL. Pellitorine (**2**), as well as compounds **3** and **4** were isolated as white powders in yields of 0.6 mg (**2**), 0.8 mg (**3**), and 0.8 mg (**4**) from 108 mg of fraction XV. Anacycline (**1**) was purified in a yield of 3.5 mg from 36 mg of fraction XIX. From a 120 mg of fraction XX, 9.2 mg of (**5**), 2.2 mg of (**6**) and 12.3 mg of anacycline (**1**) were obtained. A yield of 7.8 mg of compound (**7**) could be purified from a 72 mg of fraction XXII. Furthermore, compounds **8** and **9** were isolated as mixture (1:4) in a yield of 21.1 mg from a 72 mg of fraction XXII. The compounds isolated from fraction XXII were obtained as yellowish-white powders.

#### 3.2.5. Analytical Data

*Tetradeca-2E,4E-dien-8,10-diynoic acid isobutylamide* (anacycline, **1**). UHPLC/+ESI-QqTOF MS: Rt 7.42 min, MS (*m*/*z*): 272.2046 (calcd. for C_18_H_26_NO^+^: 272.2009); ^1^H-NMR (600 MHz, CDCl_3_; δ (ppm), mult., *J* (Hz)): 7.18 (dd, 11,15; H-3); 6.19 (dd, 11, 15; H-4); 6.07 (dt, 7, 15; H-5); 5.79 (d, 15; H-2); 5.48 (br s; NH); 3.17 (t (2H), 6, 7; H-2′); 2.38 (m (4H); H-6, H-7); 2.23 (t (2H), 7; H-12); 1.80 (sept, 7; H-3′); 1.55 (tq, (2H), 7; H-13); 0.98 (t (3H), 7; H-14); 0.93 (d (6H), 7; H-4′, H-5′).

*Deca-*2*E,4E-dienoic acid isobutylamide* (pellitorine, **2**). UHPLC/+ESI-QqTOF MS: Rt 7.26 min, MS (*m*/*z*): 224.2022 [M + H]^+^; calcd. for C_14_H_26_NO^+^: 224.2009; ^1^H-NMR (600 MHz, CDCl_3_; δ (ppm), mult., *J* (Hz)): 7.19 (dd, 10, 15; H-3); 6.13 (dd, 10, 15; H-4); 6.07 (dt, 7; 15; H-5); 5.74 (d, 15; H-2); 5.43 (br s; NH); 3.17 (dd (2H), 6; H-2′); 2.14 (dt, (2H), 7, 7; H-6); 1.80 (sept, 7; H-3′); 1.42 (tt, (2H), 7, 7; H-7); 1.28 (m (4H); H-8, H-9 overlapping signals); 0.93 (d (6H), 7; H-4′, 5′); 0.89 (t (3H), 7; H-10).

*Deca-2E,4E,9-trienoic acid isobutylamide* (9,10-dehydropellitorine, **3**). UHPLC/+ESI-QqTOF MS: Rt 6.72 min, MS (*m*/*z*): 222.1855 [M + H]^+^; calcd. for C_14_H_24_NO^+^: 222.1855; ^1^H-NMR (600 MHz, CDCl_3_; δ (ppm), mult., *J* (Hz)): 7.19 (dd, 10, 15; H-3); 6.14 (dd, 10, 15; H-4); 6.06 (dt, 15, 7; H-5); 5.81 (dt, 7, 10; H-9); 5.75 (d, 15; H-2); 5.44 (br s; NH); 5.01 (ddt, 2, 2, 17; H-10 trans); 4.96 (ddt, 1, 2, 10; H-10 cis); 3.17 (t (2H), 6; H-2′); 2.17 (dt (2H), 7, 7; H-6); 2.06 (dt (2H) 7,7; H-8); 1.80 (sept, 7; H-3′); 1.52 (m (2H), 7; H-7) overlapping signals; 0.93 (d (6H), 7; H-4′, H-5′); ^13^C-NMR (150 MHz, CDCl_3_; δ (ppm), mult.: 168.1, (s, C-1); 142.8 (d, H-5); 141.4 (d, C-3); 138.5 (d, C-9); 128.7 (d. C-4); 122.0 (d, C-2); 115.0 (t, C-10); 47.1 (t. C-2′); 33.3 (t, C-8); 32.4 (t, C-6); 28.8 (d, C-3′); 28.1 (t, C-7); 20.3 (q, C-4′ + C-5′); All assignments confirmed by HSQC and HMBC experiments.

*Deca-2E,4E-dienoic acid 2-phenylethylamide* (**4**). UHPLC/+ESI-QqTOF MS: Rt 7.67 min, MS (*m*/*z*): 272.2026 [M + H]^+^; calcd. for C_18_H_26_NO^+^: 272.2009; ^1^H-NMR (600 MHz, CDCl_3_; δ (ppm), mult., *J* (Hz)): 7.31 (t (2H) 7; H-3′′, H-5′′); 7.23 (t, 7; H-4′′); 7.20 (d (2H) 7; H-2′′, H-6′′); 7.18 (dd, 10, 15; H-3); 6.11 (dd, 10, 15; H-4); 6.07 (dt, 7, 15; H-5); 5.67 (d, 15, H-2); 5.40 (br s; NH); 3.60 (dt (2H), 6, 7; H-2′); 2.85 (t (2H), 7; H-3′); 2.14 (dt (2H), 7, 7; H-6); 1.41 (tt (2H), 7, 7; H-7) overlapping signals; 1.28 (m (4H); H-8, H-9) overlapping signals; 0.88 (t (3H), 7; H-10).

*Undeca-2E,4E-dien-8,10-diynoic acid isopentylamide* (**5**). UHPLC/+ESI-QqTOF MS: Rt 6.55 min, MS (*m*/*z*): 244.1732 [M + H]^+^; calcd. for C_16_H_22_NO^+^: 244.1696; ^1^H-NMR (400 MHz, CDCl_3_; δ (ppm), mult., *J* (Hz)): 7.17 (dd, 11, 15; H-3); 6.19 (dd, 11, 15; H-4); 6.04 (dt, 7, 15; H-5); 5.78 (d, 15, H-2); 5.48 (br s; NH); 3.35 (dt (2H), 6, 7; H-2′); 2.38 (m, (4H); H-6, H-7); 1.98 (s; H-11); 1.63 (sept, 7; H-4′); 1.42 (dt (2H), 8, 7; H-3′); 0.92 (d (6H), 7, H-4′, H-5′).

*Tetradeca-2E,4E,12Z-trien-8,10-diynoic acid isobutylamide* (**6**). UHPLC/+ESI-QqTOF MS: Rt 7.23 min, MS (*m*/*z*): 270.1881 [M + H]^+^; calcd. for C_18_H_24_NO^+^: 270.1852; ^1^H-NMR (400 MHz, CDCl_3_; δ (ppm), mult., *J* (Hz)): 7.19 (dd, 11, 15; H-3); 6.21 (dd, 11, 15; H-4); 6.12 (dq, 7, 11; H-13); 6.08 (dt, 6, 15; H-5); 5.80 (d, 15, H-2); 5.50 (dt, 11, 1; H-12); (5.47 (br s, NH); 3.17 (dd (2H), 6, 7; H-2′); 2.46 (dt (2H), 6, 7; H-6); 2.41 (t, (2H) 7; H-7); 1.91 (dd (3H) 7, 2; H-14); 1.80 (sept, 7, H-3′); 0.93 (d (6H), 7, H-4′, H-5′). 

*Dodeca-2E,4E-dienoic acid 4-hydroxy-2-phenylethylamide* (**7**). UHPLC/+ESI-QqTOF MS: Rt 7.66 min, MS (*m*/*z*): 316.2273 [M + H]^+^; calcd. for C_20_H_30_NO_2_^+^: 316.2271; ^1^H-NMR (600 MHz, CDCl_3_; δ (ppm), mult., *J* (Hz)): 7.18 (dd, 10, 15; H-3); 7.03 (d (2H) 8; H-2′′, H-6′′); 6.79 (d (2H) 8; H-3′′, H-5′′); 6.10 (dd, 10, 15; H-4); 6.06 (dt, 7, 15; H-5); 5.68 (d, 15; H-2); 5.52 (br s; NH); 3.56 (dt (2H), 7, 7; H-2′); 2.76 (t, (2H) 7, H-3′); 2.13 (dt, 7, 7; H-6); 1.40 (tt, 7, 7; H-7); 1.27 (m (8H); H-8, H-9, H-10, H-11) overlapping signals; 0.92 (d (6H), 7, H-4′, 5′); 0.88 (t (3H), 7, H-12).

*Undeca-2E,4E-dien-8,10-diynoic acid 2-phenethylamide* (**8**). UHPLC/+ESI-QqTOF MS: Rt 6.53 min, MS (*m*/*z*): 278.1542 (calcd. for C_19_H_20_NO^+^: 278.1539); ^1^H-NMR (600 MHz, CDCl_3_; δ (ppm), mult., *J* (Hz)): 7.31 (t (2H) 7; H-3′′, H-5′′); 7.23 (t, 7; H-4′′); 7.20 (d (2H) 7; H-2′′, H-6′′); 7.18 (dd, 11, 15; H-3); 6.17 (dd, 11, 15; H-4); 6.06 (m [dt]; H-5); 5.56 (br s; NH); 3.61 (dt (2H), 6, 7; H-2′); 2.85 (t (2H), 7; H-3′); 2.38 (m (4H); H-6, 7); 1.98 (s; H-11).

*Deca-2E,4E-dienoic acid 4-hydroxy-2-phenylethylamide* (**9**) UHPLC/+ESI-QqTOF MS: Rt 6.66 min, MS (*m*/*z*): 288.1973 [M + H]^+^; calcd. for C_18_H_26_NO_2_^+^: 288.1958; ^1^H-NMR (600 MHz, CDCl_3_; δ (ppm), mult., *J* (Hz)): 7.19 (dd, 10, 15; H-3); 7.02 (d (2H) 8; H-2′′, H-6′′); 6,80 (d (2H) 8; H-3′′, H-5′′); 6.10 (dd, 10, 15; H-4); 6.06 (m [dt]; H-5); 5.69 (d, 15, H-2); 5.56 (br s; NH); 3.55 (dt (2H), 6, 7; H-2′); 2.76 (t (2H), 7; H-3′); 2.13 (dt, (2H) 7, 7; H-6); 1.41 (tt (2H), 7; H-7); 1.29 (m (4H); H-8, H-9); 0.88 (t (3H), 7, H-10). 

All analytical data were in full agreement with literature data: compounds **1**, **5**, **6** [11], **2**, **8** [12], **4** [13], **7**, **9** [15].

### 3.3. In Vitro Assays and IC_50_ Determination

Tests for antiprotozoal activities were carried out using established standard protocols at the Swiss Tropical and Public Health Institute (Swiss TPH, Basel, Switzerland). The assays and the IC_50_ determinations were performed essentially as described previously [17]. The compounds used as positive controls in the various bioassays (see Table 1) were of commercial origin, with the exception of melarsoprol, which was a gift from the WHO. Their purity (generally >95%) was specified by the manufacturers. The purity of test compounds was assessed by UHPLC/MS analyses and found to be >90% in case of compounds **1**–**6** and >80% in case of **7**. Compounds **8** + **9** represented a mixture in a ratio of approximately 1:4 (Figure S10).

## 4. Conclusions

The present study adds to the knowledge on the alkamides in roots of *A. pyrethrum*, a medicinal drug used since ancient times. Compounds **3**, **4**, and **5** are reported as constituents of *A. pyrethrum* for the first time. Compound **3**—deca-2*E*,4*E*,9-trienoic acid isobutylamide (9,10-dehydropellitorine)—is a hitherto-undescribed natural product. In the case of compound **6**, the configuration of the 12,13-double bond, which had previously been reported as *E* (although without support by any data), could be unambiguously assigned as *Z*. The reported ethnomedicinal use of *A. pyrethrum* as an antimalarial might be explained by the activity of its lipophilic constituents, as the dichloromethane extract presented moderate in vitro activity against *Plasmodium falciparum*, which is in the same range as that of the most active alkamides isolated, namely pellitorine (**2**) and dodeca-2*E*,4*E*-dienoic acid 4-hydroxy-2-phenylethylamide (**7**), of which the latter also presented considerable toxicity against mammalian cells. However, the overall antiparasitic activity is low and does not warrant further exploitation.

## Figures and Tables

**Table 1:** In vitro antiprotozoal and cytotoxic activity (IC_50_ values in μg/mL; values in μM of pure compounds are reported in brackets) of the dichloromethane (DCM) extract from *Anacyclus pyrethrum* roots and of the isolated alkamides (**3**–**5**). Selectivity indices (SIs) represent the ratio of cytotoxic over antiprotozoal IC_50_ values. Data are means of two independent determinations ± deviation from mean value. The in vitro activity data of **1** (anacycline) and **2** (pellitorine) was previously reported by us [2].

Sample	*Tbr* ^1^	*Tc* ^2^	*Ld* ^3^	*Pf* ^4^	*Cytotox* ^5^	SI *Tbr*	SI *Ld*	SI *Pf*
Pos. contr.	0.003 ± 0.001	0.439 ± 0.094	0.127 ± 0.052	0.080 ± 0.003	0.008 ± 0.001			
	(0.008)	(1.688)	(0.312)	(0.250)	(0.019)			
DCM extr. ^6^	>10	8.83 ± 0.75	4.22 ± 1.57	3.04 ± 0.07	13.4 ± 3.2	>1.3	3.2	4.4
**3**	2.91 ± 0.21	39.9	4.77 ± 1.02	7.13 ± 0.63	16.7 ± 0.1	5.7	3.5	2.3
	(13.2)	(181)	(21.6)	(32.2)	(75.3)			
**4**	4.03	5.02 ± 0.05	2.97 ± 0.54	>5	7.88	2.0	2.7	>1.6
	(14.9)	(18.5)	(11.0)	(>18)	(29.1)			
**5**	4.59 ± 0.39	16.3 ± 0.3	4.04 ± 0.71	10.3 ± 1.4	28.2 ± 6.9	6.2	7.0	2.7
	(18.9)	(66.9)	(16.6)	(42.5)	(116)			
**6**	6.37 ± 0.94	38.8 ± 2.1	5.04 ± 1.17	7.19 ± 0.49	19.4 ± 1.7	3.1	3.9	2.7
	(23.7)	(144)	(18.7)	(26.7)	(72.2)			
**7**	2.26 ± 0.18	1.88	4.19 ± 1.64	3.18 ± 0.20	0.19 ± 0.05	0.1	<0.1	<0.1
	(7.17)	(5.97)	(13.3)	(10.1)	(0.69)			
**8** + **9** (1:4)	1.66 ± 0.12	3.90	5.31 ± 0.53	7.64 ± 2.57	2.47 ± 0.36	1.5	0.5	0.3

^1^ *T. b. rhodesiense* (STIB 900 strain, bloodstream trypomastigotes, positive control: melarsoprol); ^2^ *T. cruzi* (Tulahuen C4 strain; intracellular amastigotes, pos. contr.: benznidazole); ^3^ *L. donovani* (MHOM-ET-67/L82 strain, axenic amastigotes, pos. contr.: miltefosine); ^4^ *P. falciparum* (NF54 IEF, pos.: chloroquine); ^5^ Cytotoxicity (L6 rat skeletal myoblast cell line, pos.contr.: podophyllotoxin); ^6^ Data for the crude extract were previously reported and are repeated here for comparison [2].

**Table 2:** Column chromatography (CC) fractions of *A. pyrethrum* L. (CH_2_Cl_2_ extract).

Fractions and Combined Eluates *	Elution Volume (mL) **	Yield (g)	Isolated Compounds
I-VIII 1–540	4455	0.71977	
IX 541–550	200	0.0921	
X 551–565	320	0.1638	
XI 566–585	300	0.0801	
XII 586–625	670	0.3187	
XIII 626–660	530	0.4814	
XIV 661–690	410	0.2181	
XV 691–720	370	0.1548	**2, 3, 4**
XVI 721–819	1020	0.3595	
XVII 820–890	980	0.2426	
XVIII 891–909	330	0.0696	
XIX 910–940	510	0.1803	**1**
XX 941–1010	760	0.3141	**1, 5, 6**
XXI 1011–1080	840	0.5080	
XXII 1081–1140	850	0.5072	**8, 9, 10**
XXIII 1141–1200	710	0.1973	
XXIV 1201–1210	83	0.0244	
XXV 1211–1225	120	0.0381	
XXVI 1226–1280	490	0.1175	
XXVII 1281–1319	340	0.0619	
XXVIII 1320–1335	230	0.0493	
XXIX 1336–1380	780	0.1580	
XXX 1381–1410	480	0.1136	
XXXI 1411–1470	950	0.1796	
XXXII 1471–1750	4420	0.5834	
XXXIII 1751–1830	1230	0.0782	
XXXIV 1831–1875	540	0.0508	
XXXV 1876–1885	80	0.7643	
XXXVI 1886–1919	520	0.3215	
XXXVII 1920–1959	800	0.1256	
XXXVIII 1960–2023	1080	0.1762	
	Total:	Total:	
	25,398	7.4498	

* tube 1–459: 10 mL; tube 460–2023: 20 mL; ** eluate amounts determined volumetrically.

## Supplementary Materials

Supplementary materials are available online. The NMR and mass spectra of all isolates are provided as supplementary Figures S1-SXY available online.
