# Supplementary material for: Alkamides from Anacyclus pyrethrum L. and Their in Vitro Antiprotozoal Activity
Source: Molecules. 2017 May 12;22(5):796. doi: 10.3390/molecules22050796 (PMC6154598; doi:10.3390/molecules22050796)
Supplement: Supplementary file 1 [file molecules-22-00796-s001.pdf]

Supplementary Material

Alkamides from *Anacyclus pyrethrum* L. and their *in vitro* antiprotozoal activity†

Julia B. Althaus[1,†], Claudine Malyszek[1,†], Marcel Kaiser [2,3], Reto Brun [2,3] and Thomas J. Schmidt [1,*]

[1] Institut für Pharmazeutische Biologie und Phytochemie (IPBP), University of Münster, PharmaCampus, Corrensstraße 48, Münster D-48149, Germany; E-Mail: julia.althaus@uni-muenster.de (J.B.A.)
[2] Swiss Tropical and Public Health Institute (Swiss TPH), Socinstraße 57, Basel CH-4002, Switzerland; E-Mails: marcel.kaiser@unibas.ch (M.K.); reto.brun@unibas.ch (R.B.)
[3] University of Basel, Petersplatz 1, Basel CH-4003, Switzerland

† Part of doctoral thesis of J. B. A. and MSc thesis of C. M.

* Author to whom correspondence should be addressed; E-Mail: thomschm@uni-muenster.de;
Tel.: +49-251-83-33378; Fax: +49-251-83-38341.

Received: date; Accepted: date; Published: date

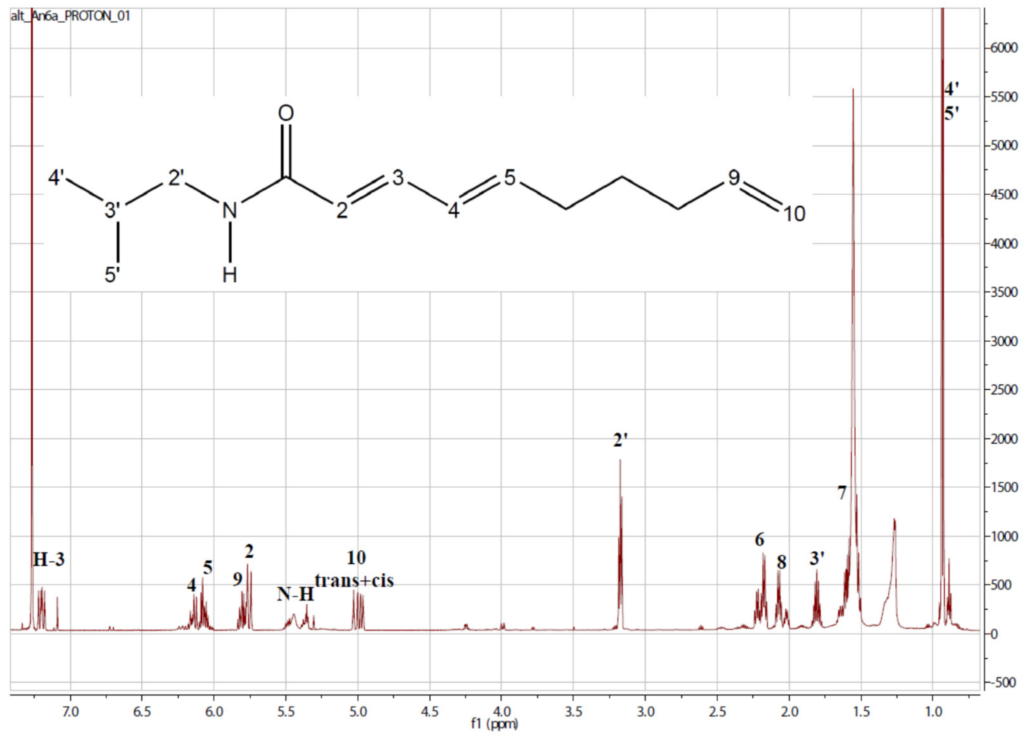

Figure S1 ^{1}H NMR spectrum of compound **3** ($CDCl_3$, 600 MHz).

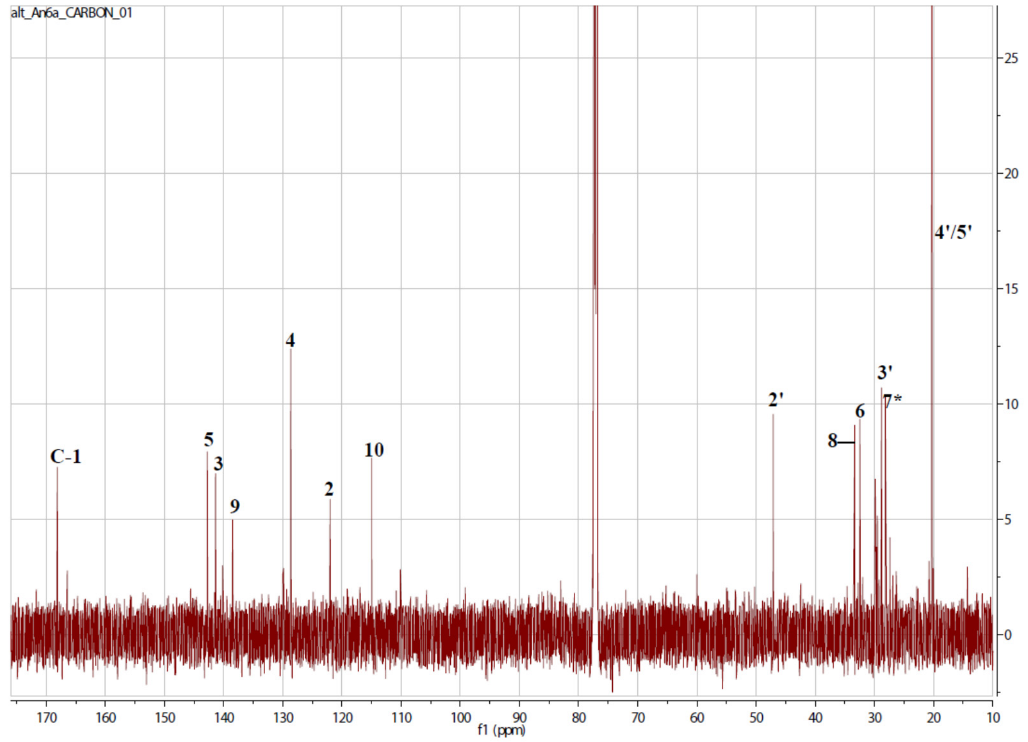

Figure S2 ^{13}C NMR spectrum of compound **3** ($CDCl_3$, 150 MHz)

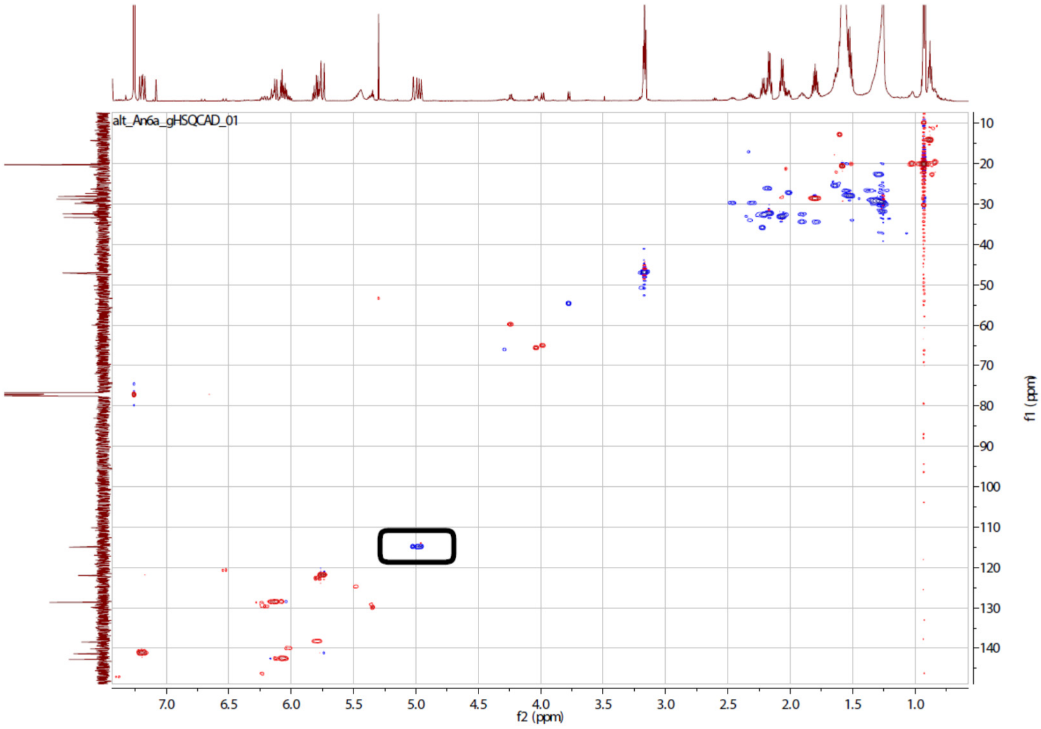

Figure S3 $^{1}H/^{13}C$ HSQC NMR spectrum of compound **3** in ($CDCl_3$, 600 MHz). Figure A5 shows a magnification of the marked area.

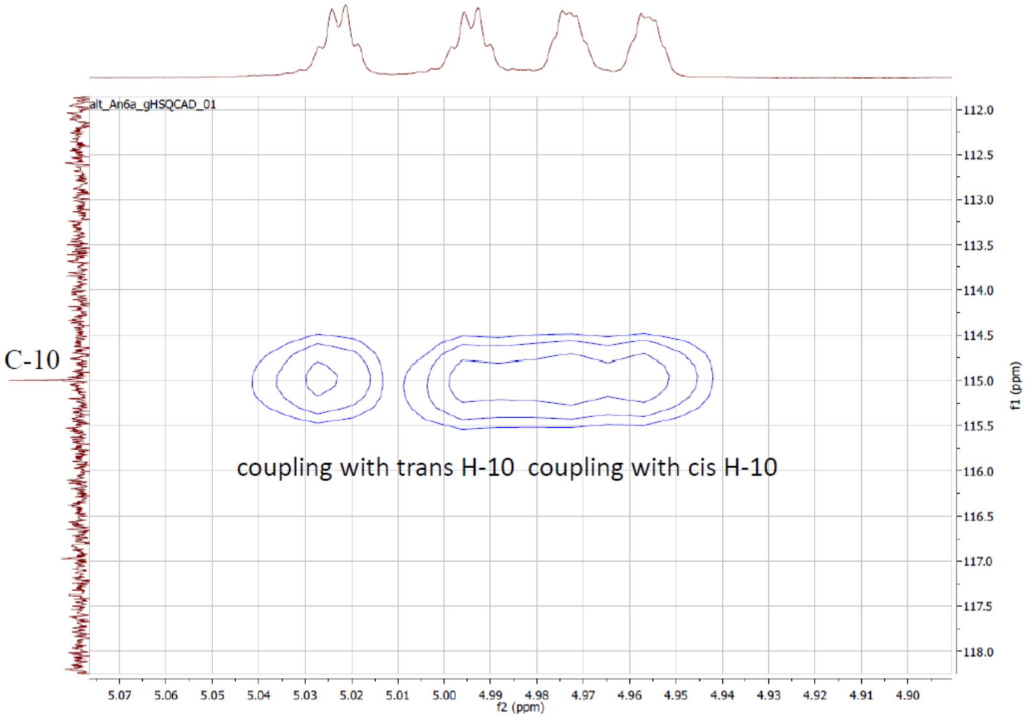

Figure S4 Extracted section of the $^{1}H/^{13}C$ HSQC NMR spectrum of compound **3** (compare Figure A3).

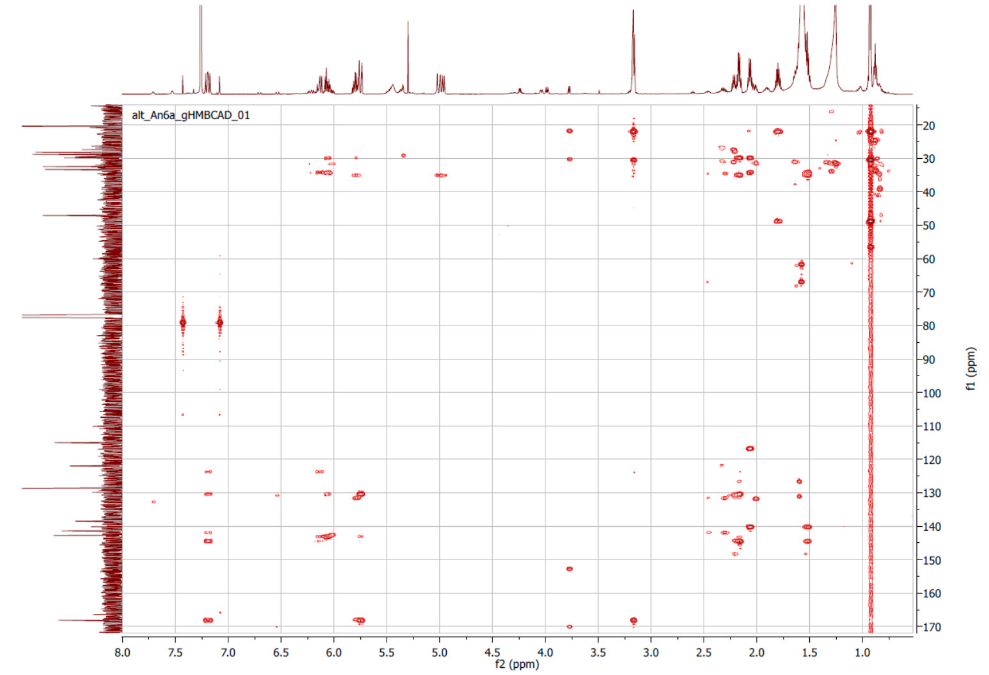

Figure S5 $^{1}H/^{13}C$ HMBC NMR spectrum of compound **3** in ($CDCl_3$, 600 MHz).

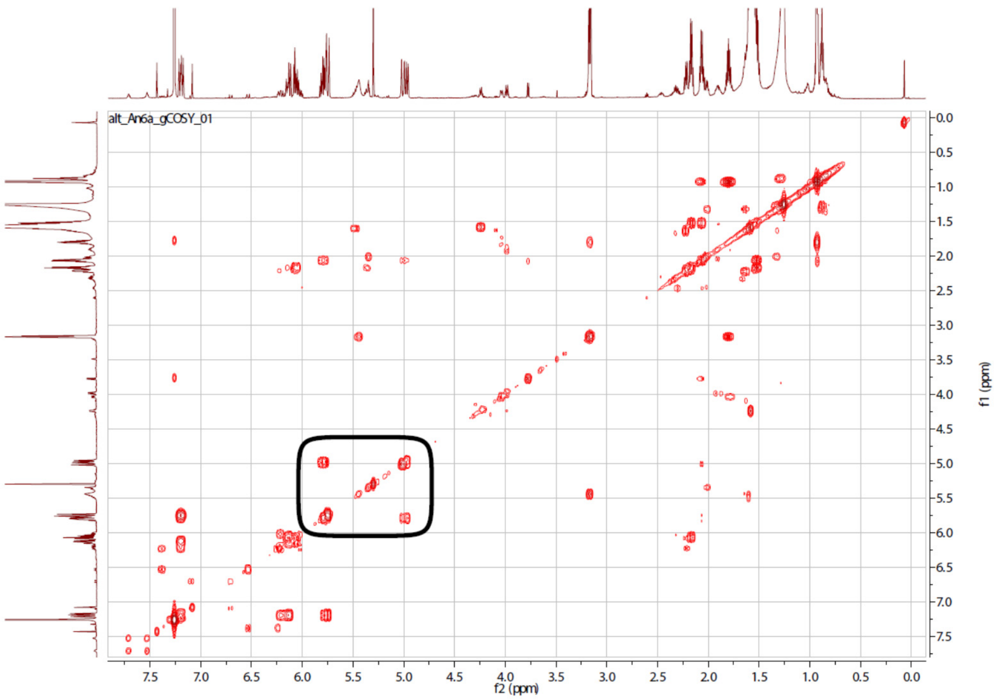

Figure S6 $^{1}H/^{1}H$ COSY NMR spectrum of compound **3** ($CDCl_3$, 600 MHz). Figure A7 shows a magnification of the marked area.

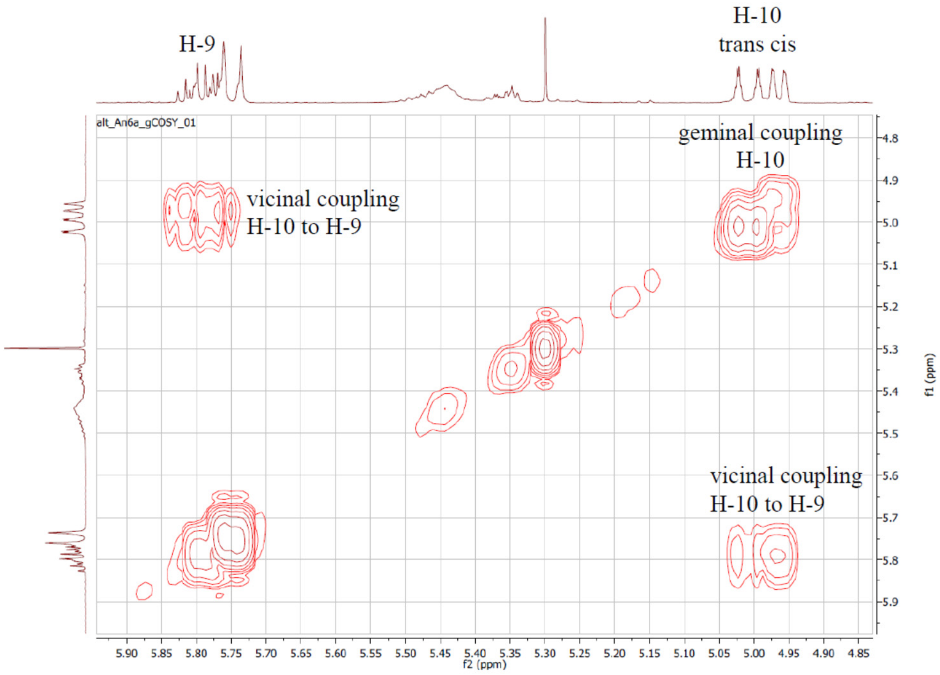

Figure S7 Extracted section of the $^1H/^1H$ COSY NMR spectrum of compound **3** (compare Figure A6).

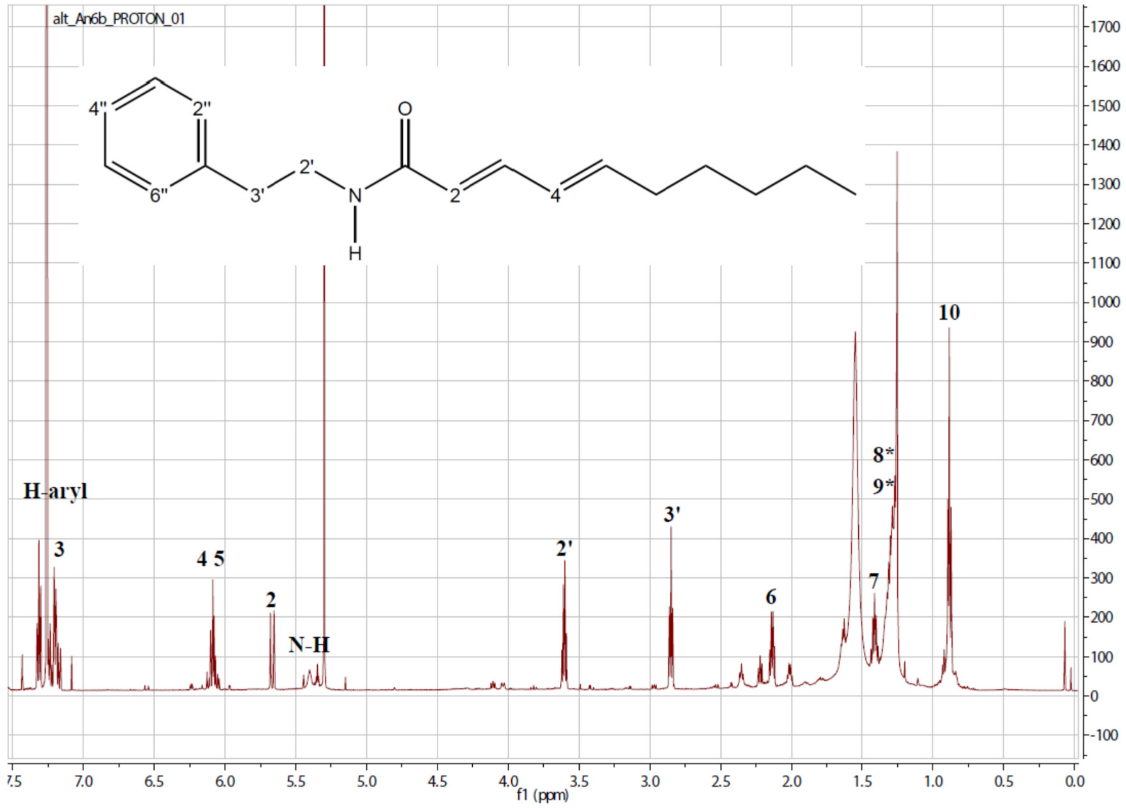

Figure S8 ^1H NMR spectrum of compound **4** ($CDCl_3$, 600 MHz).

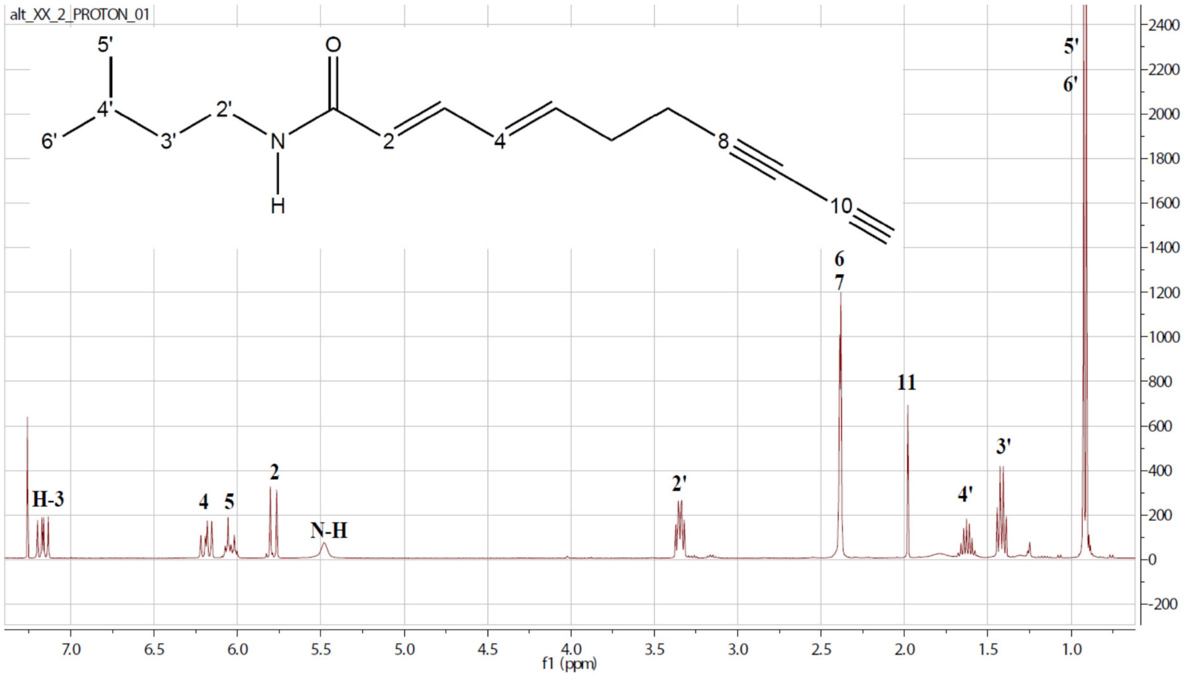

Figure S9 ^{1}H NMR spectrum of compound **5** ($CDCl_3$, 400 MHz).

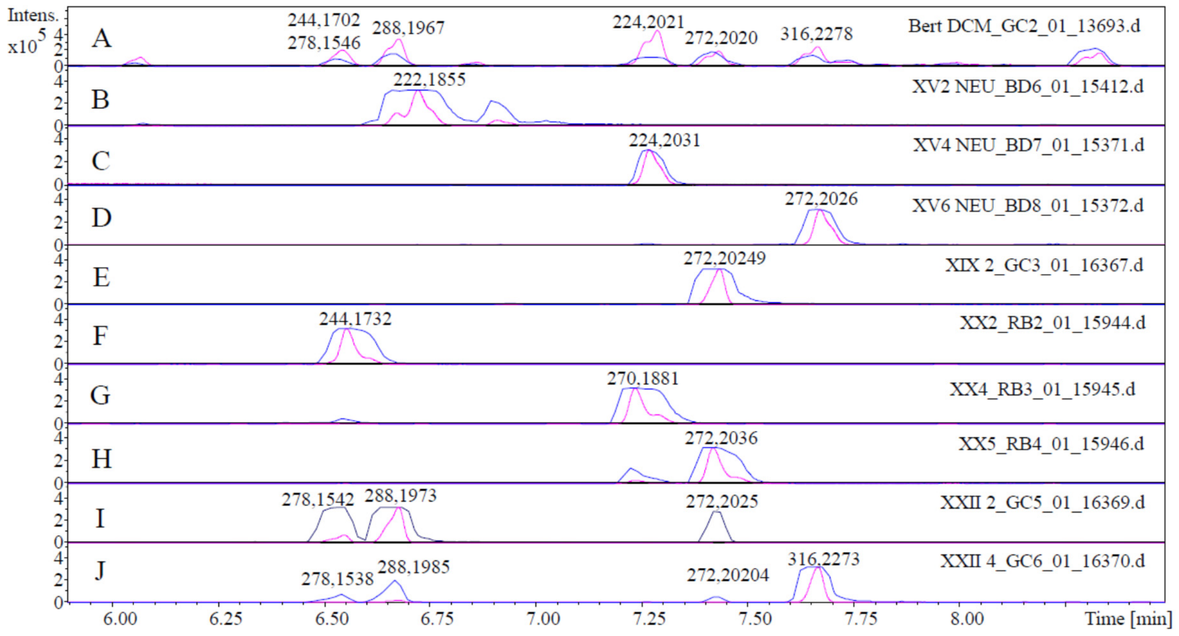

Figure S10 UHPLC/+ESI QqTOF MS-MS chromatogram of the dichloromethane extract of *A. pyrethrum* L. ((A) c= 10 mg/mL) and of the isolated alkamides ((B-J) c= 0.1 mg/mL; except (I) c= 0.2 mg/mL) from *A. pyrethrum* L.: (B) compound **3**, (C) compound **2**, (D) compound **4**, (E) compound **1**, (F) compound **5**, (G) compound **6**, (H) compound **1**, (I) compound **8** und **9** [1:4] with a small amount of **1**, (J) compound **7** (impure). Blue: Base peak chromatogram at m/z 100-500. Magenta: Base peak UV chromatogram at 260 nm.
